# Endocrinology of Hirsutism

**DOI:** 10.4103/0974-7753.66910

**Published:** 2010

**Authors:** Daisy Kopera, Elisabeth Wehr, Barbara Obermayer-Pietsch

**Affiliations:** Department of Dermatology, Internal Medicine, Medical University, Graz, Austria; 1 Department of Endocrinology, Internal Medicine, Medical University, Graz, Austria

**Keywords:** Endocrinology, hirsutism, medical treatment, polycystic ovary syndrome

## Abstract

Hirsutism represents a primary clinical indicator of androgen excess. The most common endocrine condition causing hirsutism is polycystic ovary syndrome (PCOS). Diagnosing PCOS is not easy as the signs and symptoms are heterogenous. The newest diagnostic guideline made by the Androgen Excess and PCOS Society in 2006, claims the presence of hyperandrogenism, and ovarian dysfunction (oligo / anovulation and / or polycystic ovaries). Obesity associated reproductive and metabolic dysfunctions may aggravate the symptoms of PCOS. PCOS might be underdiagnosed in non obese women because lean PCOS phenotypes might be underestimated for the syndrome. Effective medical treatment of PCOS and associated hirsutism depends on the endocrinological expertise and experience of the therapist in each individual case. An algorithm for the treatment has not been established yet.

## INTRODUCTION

Hirsutism may result from various endocrine disorders [Table 1]. Polycystic ovary syndrome (PCOS) is the most common endocrine disorder in premenopausal women (incidence 5 – 10%).[1–3] It was recognized by the Italian physician Antonio Vallisnerie, in 1721, who reported cystic ovaries in overweight infertile women. In 1921, French physicians Achard and Thiers found some coincidence between diabetes and hyperandrogenism when they coined the term ‘femmes à barbe diabetique’ (=diabetic women with beard).[4] The first systematic description mentioning amenorrhea, hirsutism, obesity, and polycystic ovaries was published by Irving Stein and Michael Leventhal, American gynecologists, in 1935. The so-called Stein-Leventhal-syndrome is a condition heavily influencing a woman’s reproductive abilities, as well as metabolic and cardiovascular health.[5]

**Table 1:** Causes of hirsutism

Frequent
Polycystic ovary syndrome (>70%)
Idiopathic hirsutism (20%)
Random
Congenital adrenal hyperplasia (due to 21-hydroxylase-deficiency)
Different types of ovarian tumors
Sertoli-leydigcell
Granulosa-thekacell
Hiluscell
Adrenal tumors
Hyperthecosis
Severe insulin resistency syndrome
Drugs
Danazol, glukokortikosteroids, penicillamin etc.
Hyperprolaktinaemia
Cushing syndrome
Akromegaly
Intersex
Gonadal dysgenesia
Pseudohermaphroditus masculinus

## DIAGNOSIS OF PCOS

**Figure 1:**
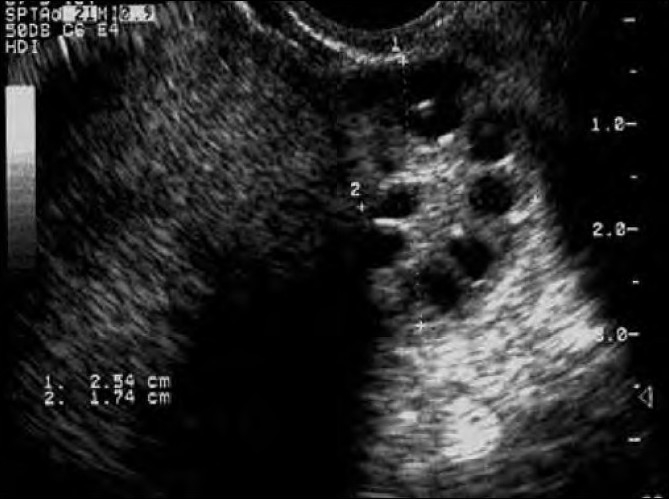
Sonographic appearance of polycystic ovaries

Diagnosing PCOS is not easy as signs and symptoms are heterogenous and vary from time to time. Diagnostic criteria have been summarized by the European Society for Human Reproduction (ESHRE) and the American Society for Reproductive Medicine (ASRM), in 2003, and denominated as ‘Rotterdam Criteria’. The presence of any two of the following three conditions corroborates the diagnosis of PCOS: oligo/anovulation, hyperandrogenism or polycystic ovaries.[Figure 1, Table 2]. All other causative diseases such as hyperprolactinemia, ‘late onset’-AGS (adrenogenital syndrome), genetic deficiency of 21-hydroxylase, Cushing’s syndrome, androgen-producing neoplasms, acromegaly, primary hypothyroidism, premature ovarian failure, and drug-associated hirsutism or dysmenorrhea have to be excluded.[5,6] Thus, we are faced with the fact that PCOS is a functional state. Polycystic ovaries per se, do not necessarily have to be present for the diagnosis of PCOS; on the other hand, polycystic ovaries alone do not justify the diagnosis of PCOS. The newest diagnostic guideline was made by the Androgen Excess and PCOS Society, in 2006; they claim the presence of hyperandrogenism and ovarian dysfunction (oligo/anovulation and/or polycystic ovaries).[7]

**Table 2:** Phenotypes of PCOS based on various symptoms and definitions (NIH, Rotterdam und AE-PCOSCriteria).[7]

Symptoms	Possible phenotypes															
	A	B	C	D	E	F	G	H	I	J	K	L	M	N	O	P
Hyperandrogenemia	+	+	+	+	–	–	+	–	+	–	+	–	–	–	+	–
Hirsutism	+	+	–	–	+	+	+	+	–	–	+	–	–	+	–	–
Oligo- or anovulation	+	+	+	+	+	+	–	–	–	+	–	–	+	–	–	–
Polyzystic ovaries	+	–	+	–	+	–	+	+	+	+	–	+	–	–	–	–
NIH 1990 criteria	√	√	√	√	√	√										
Rotterdam 2003 criteria	√	√	√	√	√	√	√	√	√	√						
AE-PCOS 2006 criteria	√	√	√	√	√	√	√	√	√							

All possible phenotypes of PCO are based on the facts of hyperandrogenaemia

Besides anovulation, limited fertility, sparse or lacking menstrual bleeding, and hyperandrogenism, PCOS may also feature dermatological conditions such as hirsutism, acne, and androgenetic alopecia, whereas, acanthosis nigricans is more common in hyperinsulinism. More than 50% of PCOS patients are obese. Obesity-associated reproductive and metabolic dysfunctions may aggravate the symptoms of PCOS. On the other hand, lean PCOS phenotypes might be underestimated for the syndrome.

## GONADOTROPHINS

**Figure 2:**
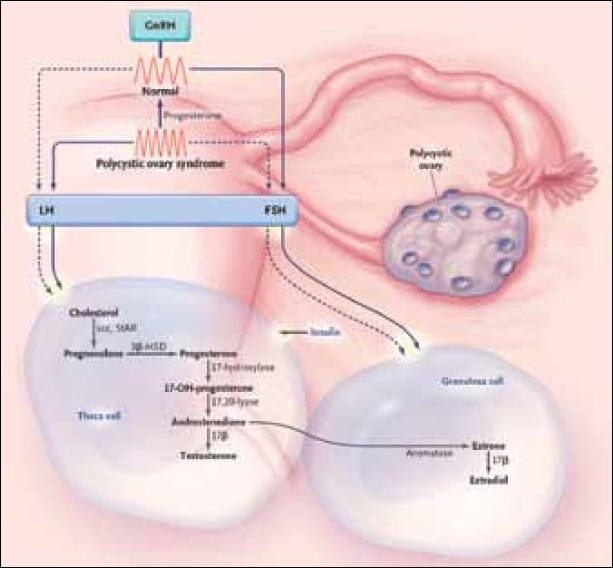
Pathogenesis of PCOS[5]

The pathogenesis of PCOS is multifactorial and depends on the biosynthesis of the steroid hormones. Triggered by the luteinizing hormone (LH) from the pituitary gland, ovarian theca cells produce androgens [Figure 2]: cytochrome P-450c17 (an enzyme with 17α-hydroxylase- and 17,20-lyase-activity) induces the production of androstendione. This is then metabolized into testosterone by 17β-hydroxysteroid dehydrogenase or into estron by aromatase. In PCOS patients, this metabolic step is in favor of testosterone production.

The Follicle-stimulating hormone (FSH) from the pituitary gland regulates aromatase-activity in granulosa cells and thereby the production of estrogens. Increased Luteinizing Hormone (LH) levels, compared to the FSH, result in increased androgen production in the ovaries.

The Gonadotropin-releasing hormone (GnRH) dominates LH/FSH secretion in the pituitary. High GnRH expression increases LH and low GnRH increases FSH synthesis. In PCOS patients the increased LH/FSH ratio relates to a high GnRH excretion, but it is still uncertain if an intrinsic malfunction of the GnRH pulse generator or relatively low levels of progesterone, as a result of anovulatory menstrual cycles, are the underlying cause [Figure 2].[5] High GnRH expression could also be caused by low pituitary progesterone sensitivity, due to low circulating androgens. This is supported by the fact that progesterone sensitivity can be increased by the therapeutic use of flutamide, an androgen receptor antagonist.[4]

In females the free androgen index (FAI) [FAI = 100× (FT/SHBG)], as a marker of PCOS, may be misleading in obese patients because obesity reduces sex hormone-binding globulin (SHBG) levels. Elevated FAI values may correlate more with obesity and less with PCOS.[8] The best biochemical marker for polycystic ovary syndrome is a raised testosterone level. Therefore, the FAI does not necessarily aid the biochemical diagnosis of PCOS.[9]

## INSULIN AND STEROID HORMONE METABOLISM

Insulin has direct and indirect influence on increased serum androgen levels (hyperandrogenism) in PCOS, stimulating the synthesis of steroid hormones in granulosa- and theca cells [Figure 2]. Insulin resistance in PCOS leads to hyperinsulinemia, which increases androgen production in the ovaries.[4] Stimulation of theca cells by insulin means an additional trigger for androgen production. At the same time insulin decreases the production of SHBG in the liver, leading to increased levels of free active testosterone. Due to hyperinsulinemia the PCO patients show increased levels of free testosterone, whereas, the total testosterone may be normal or only slightly increased.[5]

The genetic background of PCOS is not fully elucidated. An autosomal dominant trait has to be confirmed.[4] Familial predisposition seems to be the rule. Presumably the male phenotype of PCOS is represented by androgenetic alopecia.[10] There might be hormonal changes in boys with PCO mothers.[11] Another theory suspects that intrauterine retardation of growth and birth of ‘small for gestational age’ babies may show a predisposition for insulin resistance, resulting in hypertension, disturbed glucose metabolism, excess cortisol, and cortex hyperactivity in later years, provoked by too less exercise and fatty diet.[10]

## UNDESIRED HAIR GROWTH THE MAIN FEATURE OF HIRSUTISM

Hirsutism represents as a primary clinical indicator of androgen excess. It presents together with oligomenorrhea, the most common symptom of PCOS, even if the menstrual cycle appears normal. The prevalence of hirsutism in PCOS patients is 40 – 92% in European and American females. It is even more common in darker skin types, and rare in Japanese and oriental females. Almost 10% of Caucasian females feature symptoms of hirsutism that may camouflage PCOS.[12]

Hirsutism is based on a conversion of weak light vellus hair into strong dark terminal hair in androgen-sensitive body areas. Differential diagnosis of androgen-independent hypertrichosis may be crucial.

The primary androgen responsible for hair growth is dihydrotestosterone (DHT), which is synthesized from testosterone by the activity of 5α-reductase type 2. Hirsute females have increased 5α-reductase-activity in hair follicles.[12]

**Figure 3:**
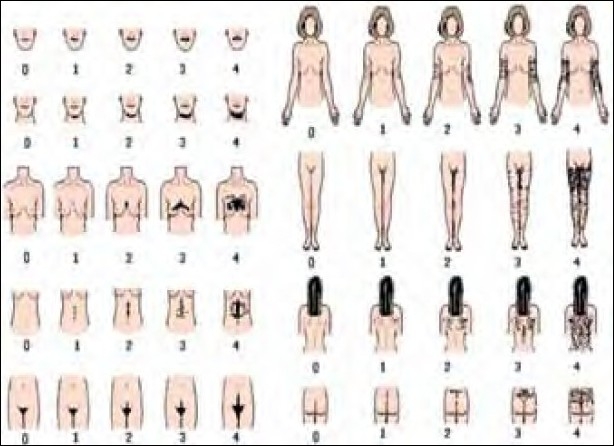
Modified Ferriman-Gallwey-score[13]

The clinical appearance of hirsutism may be quantified according to the Ferriman-Gallwey-Score, established in 1961[12,13] [Figure 3]. The phenotype also depends on ethnic factors [Table 3].

**Table 3:** Terminal hair (in %) in several body areas in hirsute women

Body area	Country and age of females					
	England (a) age 15-44 (n=257)	Holland (b) age 18-43 (n=81)	India (c) age 15-48 (n=100)	Norwayn (d) age 16-44 (n=100)	Wales (e) age 18 (n=400)	USA (f) age 18-24 (n=350)
Upper lip	41	33	0	8	26	–
Chin	10	6	0	4	–	–
Arm	18	17	3	55	–	–
Forearm	78	60	50	88	–	–
Chest	16	40	0	54	17	–
Stomac area	0	6	–	4	–	–
Belly	25	53	3	29	35	35
Upper back	0	<1	0	6	3	–
Lower back	13	7	5	24	16	–
Thigh	34	53	50	46	–	–
Calf	94	96	66	96	–	–

(a) Ferriman & Gallwey (1961); (b) Derksen *et al*. (1993); (c) Shah (1957); (d) Lunde & Grottum (1984); (e) McKnight (1964); (f) Danforth & Trotter (1922).

Hyperandrogenism, insulin-resistance, and adrenogenital syndrome represent a sub-type of PCOS that is often based on genetic predisposition. There seem to appear two types: type A which is hereditary and showing insulin resistance due to insulin receptor mutation, type B may be based on auto-immunological reasons. Both types develop antibodies against insulin receptors. Accompanying reproductive disorders appear as abnormal menstrual cycles, infertility and spontaneous abortion.[14]

## CONGENITAL ADRENAL HYPERPLASIA

Congenital adrenal hyperplasia (CAH), leads to excessive androgen production and may be diagnosed shortly after birth, in early childhood, as the adrenogenital syndrome (AGS) and ‘non-classical’ cases that occur later in puberty, denominated as ‘late onset AGS,’ present with hirsutism, irregular menstrual cycles, and / or amenorrhea. Cortisol deficiency does not occur. The underlying deficiency of 21-hydroxylase leads to overproduction of 17-OH-progesterone and androstendione.

## HYPERTHECOSIS

Hyperthecosis represents a benign condition of the ovary characterized by increased testosterone production triggered by luteinized Theca-cells. Whether this correlates with PCOS, needs to be clarified.

## SEVERE INSULIN-RESISTANCE SYNDROME

Females with severe insulin resistance in hyperinsulinemia often develop hirsutism due to increased androgen production in the ovaries that may be permitted by insulin-like growth factor-1 (IGF-1) receptors of the ovarian theca cells. Furthermore, insulin is able to lower SHBG serum levels leading to an increase in free testosterone in the peripheral blood.[15]

## DRUG-DEPENDENT HIRSUTISM

Testosterone, Dehydroepiandrosterone (DHEA), progesterone (e.g., danazole), corticosteroids, Adrenocorticotropic hormone (ACTH), and also non steroidal drugs (diphenylhydantoine, diazoxide, etc.) may be causes for hirsutism.[16]

## HIRSUTISM FROM THE INTERNAL MEDICAL POINT OF VIEW

Optimized treatment of hirsute women is based on a detailed knowledge of their status. History of menarche, menstrual cycle regularity, and first symptoms of hirsutism are very important for predicting its course and treatment. Family history of infertility, diabetes mellitus, complicated pregnancies, and similar disorders have to be recorded.

Severity of hirsutism may be defined according to a modified Ferriman-Gallwey-Score [Figure 3]. Patient self-assessment and independent physician assessment should be the rule. Values between 0 (= no excess hair growth) up to 4 (= extreme disturbing hair growth) in women may be evaluated. The severity of hair growth depends a good deal on the ethnic origin.

Body mass index, waist-to-hip-Ratio (WHR), and similar parameters may be of value to diagnose metabolic syndromes, cardiovascular disorders, and so on.

The required laboratory tests include androgens (testosterone, free testosterone, Dihydro Epiandrosterone (DHEAS), androstendione), Sex Hormone Binding Globulin (SHBG), Estrogen (E2), Luteinizing hormone (LH), Follicle Stimulating Hormone (FSH), Prolactin, Thyroid Stimulating hormone (TSH), 17-OH-progesterone, cortisol and Adrenocorticotropic hormone (ACTH). If there is any suspicion of metabolic syndrome insulin, C-peptide, glucose, Glycosylated Hemoglobin (HbA1c), triglycerides, cholesterol, Lipoproteins (HDL and LDL) estimations are necessary to detect insulin resistance or metabolic syndrome.

## LHRH-TESTING

The pituary gland plays a key role in the pathogenesis of PCOS. Therefore LHRH-testing with 25μg busereline i.v. may be helpful. Sixty minutes after application of busereline, the LH/FSH ratio should be measured, in order to detect hyperstimulation.

## MEDICAL TREATMENT OF PCOS-ASSOCIATED HIRSUTISM

Diet and correction of body mass index, lifestyle intervention, insulin sensitizers, oral contraceptives, and Vitamin D are the most common therapeutic approaches for the management of PCOS. Their application depends on the individual therapeutic aim.

Oral contraceptive pills: Estrogen–Gestagen combinations are preferably used. Estrogens lower LH levels and androgen production. Gestagens may be crucial, as they may feature androgenetic activity. Still, some of them increase the hepatogen production of SHBG–reducing, free testosterone levels. Insulin-sensitizers represent a new tool in the management of hirsutism.[5]

Antiandrogens such as cyproteronacetate CPA inhibit testosterone metabolism and are able to lower testosterone and 5α-dihydrotestosterone receptor bindings.

Spironolactone, a useful antimineralocorticosteroid, may be beneficial in PCOS.[5]

Corticosteroids may be able to lower adrenal androgens and clear the clinical features of hirsutism.[5]

Weight control is one of the first interventions in obese PCOS women, but its success is often limited, and the principle is not applicable in lean PCOS patients.

Insulin sensitizers, such as Metformin, may be helpful not only in weight loss, but they can directly stabilize insulin resistance and are able to substantially decrease androgen levels. In PCO-patients, Metformin lowers the serum levels of insulin, testosterone, and LH, resulting in decreasing body weight and normalization of the menstrual cycle.[15] There is evidence that Metformin is also beneficial in preventing early abortion and stabilizing glucose metabolism in pregnant women. However, the use in PCOS patients is off-label, due to the lack of larger studies for a Food and Drug Administration (FDA) or European Medicines Evaluation Agency (EMEA) approval.

Vitamin D supplementation is recommended in the case of 25(OH) vitamin-D insufficiency and more severe deficiency (serum levels below 30 ng/ml), which might also be helpful in the correction of dermatological and metabolic disturbances. Oral contraceptives with an established antiandrogen efficacy can help to control both androgen levels, skin symptoms, and hirsutism.

Furthermore, new therapeutic interventions might be helpful in future, such as, luteinizing hormone releasing hormone (LHRH) modulating agents, although clinical studies are currently being researched. By contrast, surgical interventions such as laparoscopic surgery have been widely used in the past and might be an alternative therapeutic option in several cases.

## CONCLUSION

In summary, PCOS is a highly frequent and often underestimated disorder in a considerable segment of hirsute women. The complex metabolic and hormonal disturbances require a concomitant interdisciplinary and individualized therapy.
